# Sacral myeloid sarcoma involving multiple metastases to the brain: A case report

**DOI:** 10.3892/etm.2015.2292

**Published:** 2015-02-13

**Authors:** SUNIMA LAMA, SU LUI, YUAN XIAO, HUAWEI ZHANG, MANDEEP KARKI, QIYONG GONG

**Affiliations:** 1 Department of Radiology, Huaxi Magnetic Resonance Research Center, Chengdu, Sichuan 610041, P.R. China; 2 Department of Orthopedics, West China Hospital of Sichuan University, Chengdu, Sichuan 610041, P.R. China

**Keywords:** magnetic resonance imaging, immunohistochemistry, myeloid sarcoma, sacral spine

## Abstract

The presentation of myeloid sarcoma (MS) in the bone is common; however, rarely does the tumor occur in the sacral spine, and in a normal patient with no history of acute myeloid leukemia. The present study describes the rare case of a previously healthy 24-year-old male patient, who presented with a history of six months of repeated pain in the right leg and hip and limping for less than a month, who was diagnosed with sacral MS. Despite receiving surgical management and chemotherapy promptly subsequent to the diagnosis and undergoing close observation following the treatment, the patient still developed metastases to multiple sites of the brain. Taking into account the similar presentation of this rare disease to other entities, the early and accurate diagnosis of MS is vital, and the condition should be considered as a threatening manifestation with the possibility of metastasis to other sites of the body.

## Introduction

Myeloid sarcoma (MS), also known as granulocytic sarcoma or chloroma, is a rare neoplastic condition that is characterized by the occurrence of one or more tumor masses, consisting of immature myeloid cells presenting at an extra-medullary site (1). MS is subclassified according to the most abundant cell type, the commonest form being the blastic variant followed by the monoblastic and myelomonocytic variants (1). Any site of the body can be affected, but the most common locations are the skin, peritoneum, lymph nodes and bone (2,3). The most common region of the spine involved is the thoracic spinal cord (64%), followed by the lumbar (29%), sacral (20%) and cervical (5%) segments (4). Spinal MS of the sacrum is not common, and spinal cord compression caused by an MS is even more rare (5–7). Most commonly, MS is found concurrently in a patient with previously or recently recognized acute myeloid leukemia (AML), but it may also precede the appearance of blood or bone marrow disease. The definitive diagnosis of a MS usually requires a biopsy of the lesion in question. Imaging examinations, such as enhanced magnetic resonance imaging (MRI), may display the exact location and size of the lesion to facilitate the early diagnosis of MS. The present study describes an uncommon case of spinal MS at the sacral level L5-S2 in a 24-year-old male who, notably, had no previous history of any blood disease. Written informed consent was obtained from the patient.

## Case report

A 24-year-old male patient was admitted to the West China Hospital of Sichuan University (Chengdu, China) on 29 March, 2012 with a history of six months of repeated pain in the right leg and hip and limping for less than a month. The patient had not initially paid attention to the symptoms but the pain later was so severe that it became difficult for him to sleep and walk normally. The symptoms were also accompanied by frequent urination and constipation and by slight urinary incontinence while sneezing. There was no past history of medical illness, including AML, or surgery.

**Figure 1:**
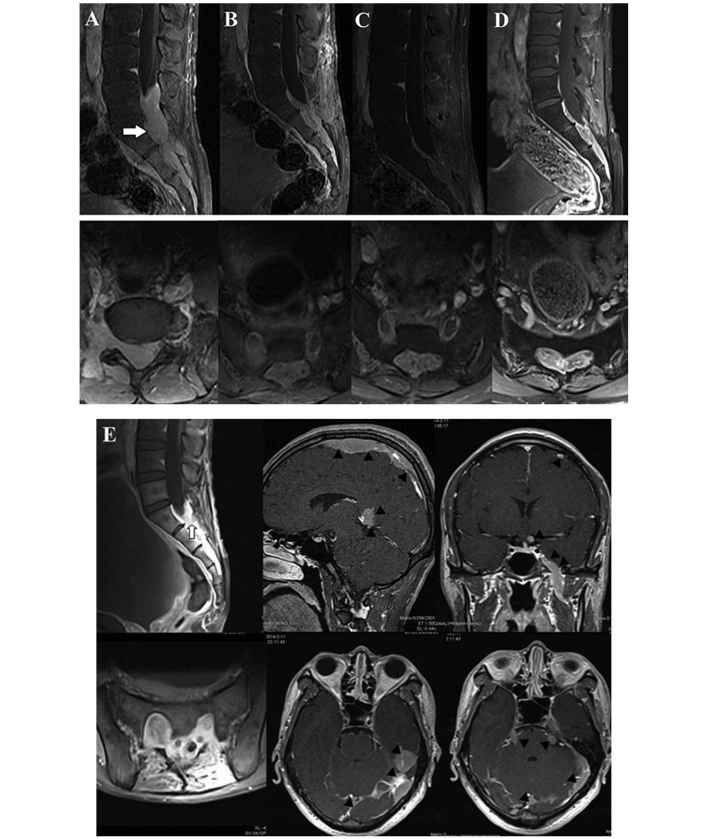
Sagittal and axial T2-weighted magnetic resonance imaging scans demonstrating the progression of the disease, including the treatment phase and the development of metastasis. (A) Sacral spinal mass at presentation extending from L5 to S2 associated with the destruction of adjacent soft tissues and bone strcutures (white arrow). (B) Post-surgery, and at the time of the first course of chemotherapy. (C) Following the second course of chemotherapy. (D) Ten months after the second course of chemotherapy. (E) Seventeen months after the second course of chemotherapy, showing metastasis to different sites of the brain (black arrowheads).

Physical examination on admission revealed slight hypoesthesia in the right hip and bilateral lower extremities. Other neurological examination results were unremarkable. MRI of the spine revealed a mass lesion within the spinal canal extending from L5 to S2. The mass had intermediate signal intensity on the T1- and T2-weighted images and showed relatively homogeneous enhancement in contrast-enhanced scans. The lesion extended from the sacral hole into the right paravertebral soft tissue. Destruction of the adjacent bone was also observed (Fig. 1A). The primary diagnosis of this case was chordoma.

**Figure 2:**
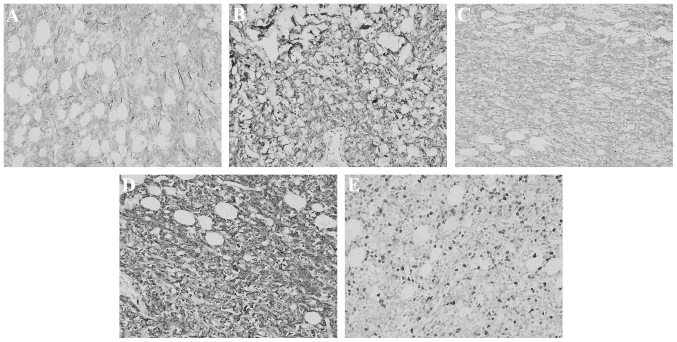
Biopsy of the sacral myeloid sarcoma. The neoplastic cells were positive for (A) CD34, (B) CD43, (C) CD99, (D) myeloperoxidase and (E) Ki67 (hematoxylin and eosin staining; magnification, ×40). CD, cluster of differentiation.

Following the preoperative examinations, the patient underwent L5-S2 decompression semi-laminectomy under general anesthesia on 31 March, 2012. The tumor was partially resected, as it was closely adherent to the dural sac and surrounding structures. Pathological material obtained at surgery revealed malignant MS. As shown in Fig. 2, immunohistochemical study of the tumor cells revealed positive staining for myeloperoxidase, cluster of differentiation (CD) 43, CD34 and CD99 and a Ki67 index of ~50%. Negative results were obtained for the following molecules: CD20 (−), CD3 (−), terminal deoxynucleotidyl transferase (−), CD20 (−), CD3 (−), CD30 (−), B-cell lymphoma (−), CD10 (−), PWWP domain-containing protein mutated melanoma-associated antigen 1 (MUM1) (−), desmin (−), myogenin (−) and neuron specific enolase (NSE) (−). The bone marrow aspirate was analyzed and the results showed acute leukemia, morphologically AML-2 with 60% myeloblasts. Lumbar puncture of the cerebrospinal fluid (CSF) showed few lymphocytes. The final histological diagnosis was therefore MS.

The patient received chemotherapy twice following the surgery with daunorubicin (70 mg/day) for three days and cytosine arabinoside (400 mg/day) for seven days, along with platelet transfusion during the second course of chemotherapy and other symptomatic treatment. Improvements were observed in the MRI following the treatment with a reduction in the size of the tumor (Fig. 1B–D); however, the MRI scans of the patient taken in February 2014 showed invasion and metastasis of the tumor to the brain with multiple nodular lesions present at different sites, such as the pituitary stalk, optic chiasm, pineal region, left tentorium, left cistern, left sigmoidal sinus, falx, bilateral trigeminal nerves, left submandibular branch of the trigeminal nerve and cavernous sinus (Fig. 1E).

## Discussion

MS is a rare tumor of immature granulocytic cells that appears concomitantly in 15–35% of cases with AML. It can affect any organ system, such as in the head, neck, bone and skin, and is less commonly found in the central nervous system (CNS) (8), particularly spinal MS associated with spinal cord compression (7,9–12). MS in the sacral region migrating towards the CNS is even more rare. The clinical symptoms depend on the size and localization of the tumor and the most common signs and symptoms that have been presented in cases of MS to date are compression signs accompanied by severe pain and abnormal bleeding (13); these symptoms are quite common for alternative diagnoses and can be misleading. In the present case, differential diagnoses of chordoma, malignant lymphoma and acute leukemia were made at the initial presentation, prior to the pathological findings and immunohistochemical staining confirming a diagnosis of MS.

The diagnosis of MS relies on a number of examinations, including bone marrow aspiration, biopsy or peripheral blood smears and immunohistochemical detection. More importantly, myelo- and/or monoblasts in the MS lesions exhibit antigenic profiles and express myeloid- and monocytoid-associated antigens, including CD13, CD14, CD33, CD64, CD68 and c-Kit (CD117), as well as lysozyme (14). The characteristic microscopic growth pattern of myeloid cells is either a diffuse or an Indian file pattern and the Ki67 index is usually high, ranging between 50 and 95%. In the present case, positive results were found for the above-mentioned antigens (as shown in Fig. 2). Staining for myeloperoxidase, which is considered to be the most useful marker for the identification of MS due to its high sensitivity and specificity to myeloid cells (15), was also positive in the current case.

In the present case, MRI was used to detect the abnormality within the spine and later estimate the efficacy of the treatment. MRI is said to be the technique of choice in diagnosing intraspinal or paraspinal pathology and is useful in distinguishing MS from other entities, i.e. hemorrhage or abscess (16), although fluorodeoxyglucose-positron emission tomography has also been used to investigate extramedullary AML (17) and granulocytic sarcoma of the sacral spine (11). A study of spinal MS found that diffuse bone marrow infiltration and intermediate signal intensity (less high signal intensity) of MS on T2-weighted images is usually helpful (18). The imaging characteristics of MS are often described as hypointense or isointense on T1-weighted MRI and heterogeneously isointense or hyperintense on T2-weighted MRI, with homogeneous enhancement following contrast injection (19). The imaging results of the present case were consistent with those of these previous studies, showing isointense enhancement in both T1- and T2-weighted images and homogeneous enhancement in contrast-enhanced MRI scans.

Approximately two years after the diagnosis of the disease in the present patient, metastases, which may have travelled through the CSF, were found in multiple sites of the CNS; to the best of our knowledge, such an incidence has not yet been reported in any case of MS of the sacrum. Careful investigation and diagnosis are of the utmost importance in cases of MS and may have a significant impact on the treatment, survival rate and prognosis of the patient. Numerous cases of MS have been misdiagnosed as lymphoproliferative disorders, particularly lymphoma (4). Although the rate of misdiagnosis has been reduced to 25–47% (15,20), the chances of the patient receiving an incorrect diagnosis remain high. Thus, an early and precise diagnosis of spinal MS with MRI evaluation facilitates the appropriate treatment with further therapy.

The optimal treatment of MS is not yet well defined; however, the options include surgical resection, intravenous and/or intrathecal chemotherapy, radiation therapy or any combination of these treatments. A previous review showed that patients receiving systemic chemotherapy may have an advantage with 46% one-year survival as compared with 16% one-year survival in patients treated with other methods (8). To date, no strict chemotherapeutic regimen for MS has been introduced. Certain studies have suggested that stem cell transplantation may be an effective therapy for patients with MS with longer survival (13,20); however, despite these available options, the recurrence rate is high. Although the patient in the present case received surgery and induction chemotherapy, which relieved the spinal cord compression and pain of the patient to a certain extent, a relapse was recently observed, with metastasis to the brain. The prognosis of MS is controversial and has been reported to be different for isolated MS and MS concurrent with AML or extramedullary disease. Overall, it appears that MS is associated with a poor prognosis, and the mean survival time of patients with MS ranges between 2.5 and 22 months (21). The patient in the present case has surpassed this duration of time and is still alive, which again indicates the importance of the selection of the most suitable therapeutic option.

In conclusion, the present study describes a rare case of sacral MS in a previously healthy, non-leukemic patient associated with metastasis of the tumor to the brain. Although the patient has been relieved of the symptoms and the tumor mass has reduced in size, the treatment of the metastases remains to be determined. Several points can be made from the current case. Firstly, sacral MS, which presents with similar symptoms to other spinal lesions, cannot be neglected and should be kept under the differential diagnosis of spinal mass lesions. Secondly, important examinations, such as MRI, bone marrow aspiration and pathological and histological staining, can provide an accurate diagnosis if carried out promptly. Finally, although several methods have been proposed, the patient in this case received prompt surgical resection and induction chemotherapy, which may have been the factor that has led him to survive for approximately two years, to date. Routine MRI should be considered as an important investigative step for metastasis.
